# Molecular Sites for the Positive Allosteric Modulation of Glycine Receptors by Endocannabinoids

**DOI:** 10.1371/journal.pone.0023886

**Published:** 2011-08-25

**Authors:** Gonzalo E. Yévenes, Hanns Ulrich Zeilhofer

**Affiliations:** 1 Institute of Pharmacology and Toxicology, University of Zurich, Zurich, Switzerland; 2 Institute of Pharmaceutical Sciences, ETH Zurich, Zurich, Switzerland; University of Connecticut, United States of America

## Abstract

Glycine receptors (GlyRs) are transmitter-gated anion channels of the Cys-loop superfamily which mediate synaptic inhibition at spinal and selected supraspinal sites. Although they serve pivotal functions in motor control and sensory processing, they have yet to be exploited as drug targets partly because of hitherto limited possibilities for allosteric control. Endocannabinoids (ECs) have recently been characterized as direct allosteric GlyR modulators, but the underlying molecular sites have remained unknown. Here, we show that chemically neutral ECs (e.g. anandamide, AEA) are positive modulators of α_1_, α_2_ and α_3_ GlyRs, whereas acidic ECs (e.g. N-arachidonoyl-glycine; NA-Gly) potentiate α_1_ GlyRs but inhibit α_2_ and α_3_. This subunit-specificity allowed us to identify the underlying molecular sites through analysis of chimeric and mutant receptors. We found that alanine 52 in extracellular loop 2, glycine 254 in transmembrane (TM) region 2 and intracellular lysine 385 determine the positive modulation of α_1_ GlyRs by NA-Gly. Successive substitution of non-conserved extracellular and TM residues in α_2_ converted NA-Gly-mediated inhibition into potentiation. Conversely, mutation of the conserved lysine within the intracellular loop between TM3 and TM4 attenuated NA-Gly-mediated potentiation of α_1_ GlyRs, without affecting inhibition of α_2_ and α_3_. Notably, this mutation reduced modulation by AEA of all three GlyRs. These results define molecular sites for allosteric control of GlyRs by ECs and reveal an unrecognized function for the TM3-4 intracellular loop in the allosteric modulation of Cys-loop ion channels. The identification of these sites may help to understand the physiological role of this modulation and facilitate the development of novel therapeutic approaches to diseases such as spasticity, startle disease and possibly chronic pain.

## Introduction

Glycine receptors (GlyRs) are anion-selective transmitter-gated ion channels of the Cys-loop superfamily. They are critical for the control of excitability in the spinal cord, brain stem, and a few select brain areas. Diminished glycinergic inhibition plays a key role in inflammation-induced pain hypersensitivity and in heritable startle disease [1]–[3]. GlyRs are pentameric complexes composed of α and β subunits, which can form homomeric (α) or heteromeric (αβ) receptors. Each subunit possesses an amino-terminal extracellular domain (ECD), four transmembrane domains (TM) and a large intracellular loop (IL) between TM3 and TM4 [3]–[4]. Molecular cloning studies have identified four highly conserved α subunits (α_1-4_), which differ in their developmental and regional expression [4] as well as in their biophysical properties [5]–[6]. As a consequence, a growing body of evidence suggests specific roles for GlyR isoforms in diverse physiological processes [7]-[9].

GlyRs are also subject to allosteric modulation by metal ions or small organic compounds including zinc, general anesthetics and ethanol [3], [10]. Two well characterized allosteric sites are localized in the TM and ECD regions. Residues within the TM2 and TM3 domains of α_1_ GlyRs combine to form a cavity which serves as an ethanol and general anesthetic binding pocket [11]–[12], whereas several amino acids in the ECD contribute to the modulation by zinc [13]–[14]. Interestingly, the GlyR isoforms differ in their sensitivities to these modulators [15]–[17]. Recent studies have reported that the GlyR activity can be modulated allosterically by certain endocannabinoids (ECs) in a G-protein-independent manner [10], [18]-[20]. ECs are endogenous lipid signaling molecules, structurally related to arachidonic acid, that primarily, but not exclusively, act through G protein-coupled cannabinoid receptors (CB-R) [21]. Interestingly, other putative ECs and their synthetic derivatives, despite being poor CB-R activators, are able to effectively modulate ion channels [22]–[25]. ECs which modulate GlyRs not only spare GABA_A_-R [19], but also exhibit differential effects on GlyR subtypes [10], [20]. Importantly, the sites for the EC modulation of GlyRs have been shown to be different from the TM sites responsible for ethanol modulation [19]. By analyzing different ECs in chimeric and mutant GlyRs, we identified a group of residues that jointly determine the EC sensitivity of GlyR isoforms. These residues are localized along the N-terminal ECD, TM and IL regions and are not related to previously known allosteric sites on GABA_A_ or GlyRs. Thus, our results define hitherto unrecognized allosteric sites for ECs on GlyR, which could possibly enable the development of subtype-specific GlyR modulators.

## Results

### Allosteric modulation of GlyR by endocannabinoid derivatives

**Figure 1:**
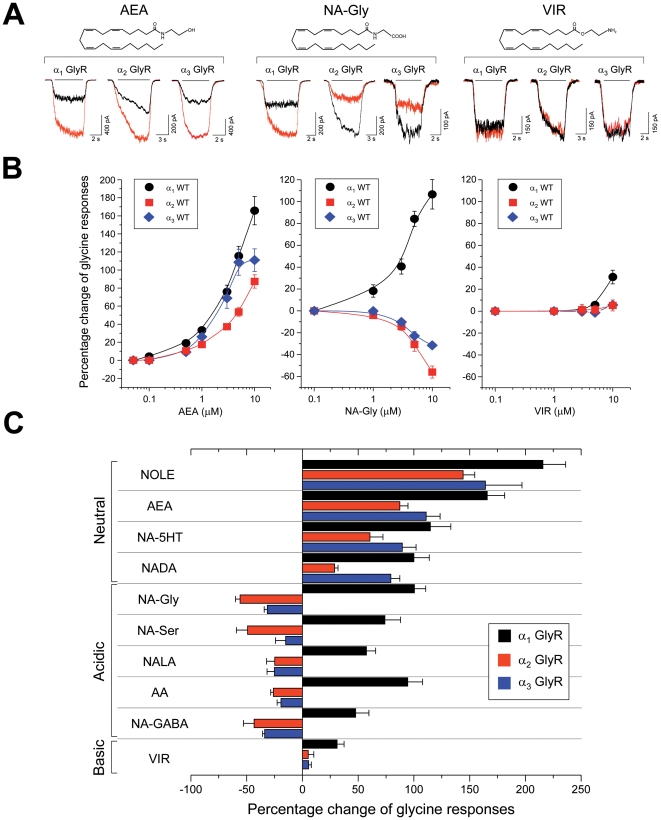
Modulation of different GlyR subtypes by ECs. (**A**) Glycine-activated membrane currents through wild-type α_1_, α_2_ and α_3_ GlyRs under control conditions (black) and in the presence of AEA, NA-Gly and VIR (red; all 10 µM). Membrane currents were activated by equipotent (EC_10_) glycine concentrations for each particular subunit. Chemical structures for the ligands are also shown. (**B**) Concentration-response curves. (**C**) Summary of the EC-mediated allosteric modulation of GlyRs subunits obtained at 10 µM concentration. Note that all acidic ECs tested still potentiated the α_1_ GlyR currents, but inhibited currents through α_2_ - α_3_ GlyRs. NOLE, noladin ether; AEA, anandamide; NA-5HT, arachidonyl serotonin; NADA, N-arachidonyl dopamine, NA-Gly; N-arachidonyl glycine; NA-GABA, N-arachidonyl-GABA; NA-Ser, N-arachidonoyl-L-serine; NALA, N-arachidonoyl-L-alanine; AA, arachidonic acid; VIR, virodhamine. Data are means ± SEM from 6-15 cells.

In a first set of experiments, we examined the sensitivity of the three most abundant GlyR α subunits (α_1_, α_2_ and α_3_) to different ECs. Most of these molecules have been identified as ECs or putative ECs from brain or spinal cord extracts [21]–[22]. We found that low micromolar concentrations of neutral and acidic ECs modulated currents through homomeric GlyRs expressed in HEK 293 cells elicited at low glycine concentrations (EC_10_) (Figure 1, Figure S1). Five of the compounds tested contained free carboxyl groups (NA-Gly, N-arachidonoyl glycine; NA-Ser, N-arachidonoyl-L-serine; NALA, N-arachidonoyl-L-alanine; NA-GABA, N-arachidonoyl-GABA; AA, arachidonic acid) and displayed subunit-specificity. For example, NA-Gly induced a significant potentiation of glycine-activated currents through α_1_ GlyRs by 101±11% (10 µM, n = 14), whereas currents through α_2_ or α_3_ GlyRs were inhibited by −56±5% (n = 14) and by −32±3% (n = 14) at 10 µM, respectively. On the other hand, the four hydroxylated, neutral compounds (NOLE, noladin ether; AEA, anandamide; NA-5HT, arachidonyl serotonin; NADA, N-arachidonyl dopamine) showed consistent positive allosteric modulation of all the GlyR isoforms, suggesting that carboxyl groups were required for the inhibition of α_2_ and α_3_ GlyRs. Interestingly, the basic EC virodhamine (VIR), which possesses an amino group instead of a hydroxyl or carboxyl group, did not significantly alter the α_2_ and α_3_ GlyR responses and behaved as a very weak α_1_ GlyR modulator (Figure 1A–C). These results suggest that hydroxyl groups on ECs are required for the positive modulation of all three GlyR isoforms, whereas carboxyl groups are structural prerequisites for the inhibition of α_2_ and α_3_ GlyRs. These findings strongly suggest that the existence of specific molecular sites in the different GlyR isoforms which underlie subunit-specific actions of ECs on the GlyR subtypes.

### Functional screening of endocannabinoid molecular sites on GlyR

**Figure 2:**
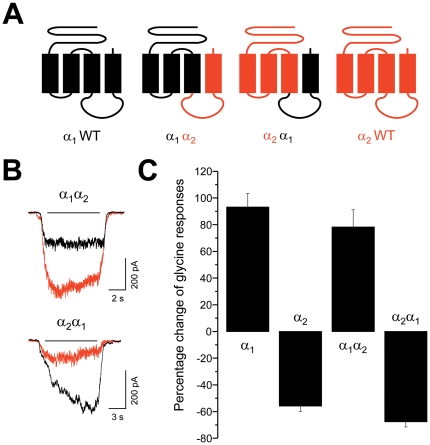
NA-Gly effects on chimeric GlyR constructs. (**A**) Schematic depiction of wild type and chimeric GlyRs. (**B**) Examples of whole-cell currents recorded from α_1_α_2_ or α_2_α_1_ GlyRs before (black) and during the application of NA-Gly (10 µM, red). (**C**) Percent change of the normalized glycinergic membrane currents during the application of NA-Gly (10 µM) using equipotent (EC_10_) glycine concentrations. The exchange of the IL between TM3 and TM4 plus the TM4 domain between α_1_ and α_2_ GlyRs did not significantly influence the NA-Gly-induced modulation.

We next aimed at determining the molecular mechanisms and elements underlying the differential allosteric modulation of GlyRs by acidic ECs. To this end, we focused on NA-Gly and examined its actions at different glycine concentrations (Figure S2). Interestingly, the potentiation elicited by NA-Gly on α_1_ GlyRs at EC_10_ of glycine was significantly attenuated at a higher glycine concentration (EC_50_), whereas the NA-Gly-induced inhibition of α_2_ and α_3_ GlyRs remained unaltered. These results suggest that the mechanisms and molecular sites involved in potentiation and inhibition are different and non-conserved between subunits. In order to define the molecular sites involved, we next examined the NA-Gly effects using a set of chimeric and mutant GlyRs. To analyze the importance of TM4 and the IL domains, we first studied a pair of chimeric GlyR constructs in which the regions upstream of the IL between α_1_ and α_2_ were exchanged (Figure 2A). We found that this exchange did not significantly affect the modulation by NA-Gly (Figure 2B–C). Based on these results, we can conclude that IL and TM4 domains do not significantly contribute to the subunit-specific modulation by NA-Gly.

**Figure 3:**
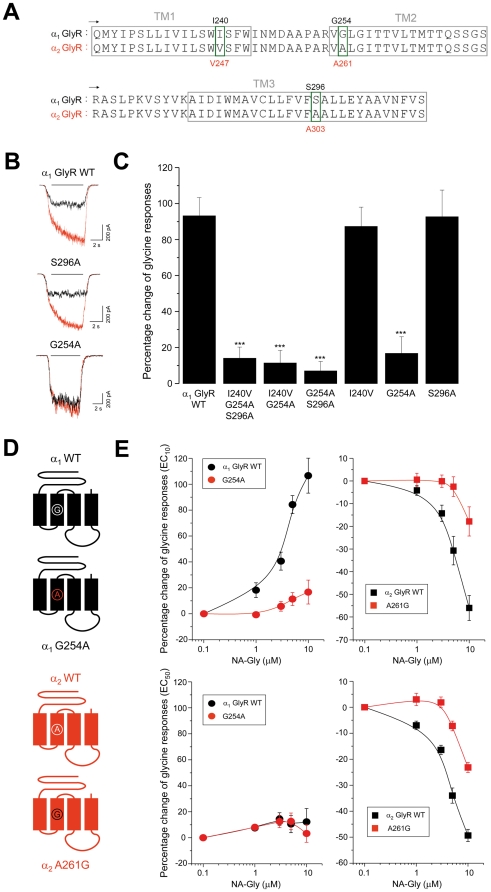
Positive and negative NA-Gly allosteric effects on α_1_ and α_2_ GlyRs are influenced by a single TM2 domain residue. (**A**) Primary sequence alignment between α_1_ and α_2_ GlyR subunits from TM1 to TM3 regions. The 3 non-conserved residues are highlighted by green boxes. (**B**) Examples of current traces through GlyRs with mutated TM domains, in absence (black) or presence of NA-Gly (red) (**C**) The bar graphs summarizes the normalized glycine-evoked current enhancement after the application of 10 µM NA-Gly on α_1_ GlyRs with mutations in specific residues within the TM domains (**D**) Schematic representation of wild-type and TM2-mutated GlyRs (**E**) Concentration-response curves for NA-Gly in wild-type and TM-mutated α_1_ and α_2_ GlyRs using two different agonist concentrations. Note that the specific mutation G254A in α_1_ GlyRs significantly attenuated the EC potentiation, whereas the reverse TM2 mutation in α_2_ GlyRs (A261G) additionally decreased the NA-Gly-induced inhibition.

Next, we evaluated the importance of TM regions upstream from the IL. Previous studies have shown that TM regions of GABA_A_ and GlyRs contain critical residues for several allosteric modulators, such as ethanol, general anesthetics and neurosteroids [11]–[12], [26]–[27]. Furthermore, very recent evidences have been reported that TM residues are critical for the potentiation of recombinant α_1_ GlyRs by Δ^9^-tetrahydrocannabinol (THC) and cannabidiol, two phytocannabinoids [28]–[29]. Thus, a rational explanation for the NA-Gly subunit-specific modulation could arise from differences in TM domain composition. An amino acid sequence alignment of the TM regions of α_1_ and α_2_ GlyR subunits revealed only 3 non-conserved residues: I240, G254 and S296 in α_1_ GlyRs or A247, A261 and A303 in α_2_ GlyRs (Figure 3A). To investigate the contribution of these residues, we examined the NA-Gly-induced potentiation of triple, double and single mutant α_1_ GlyRs in which these amino acids have been substituted with their α_2_ GlyR counterparts. Importantly, these mutated receptors as well as all others described in this study responded normally to glycine application (Table 1). Mutation of these non-conserved amino acids (I240V/G254A/S296A) in α_1_ GlyR significantly reduced the GlyR current potentiation by NA-Gly to 14±6% (10 µM, n = 7). Subsequent analyses using single and double mutated α_1_ GlyRs demonstrated that the G254A mutation reduced the NA-Gly modulation similarly (17±9%, 10 µM, n = 8), indicating that G254 is a critical determinant of α_1_ GlyR potentiation (Figure 3B-D). At higher agonist concentrations, no differences were found between wild type and G254A α_1_ GlyRs. On the other hand, the reverse A261G mutation in α_2_ GlyRs significantly attenuated NA-Gly-induced inhibition at low and high glycine concentrations (Figure 3D), indicating a role for this residue in both positive and negative modulation of α_1_ and α_2_ GlyRs by NA-Gly. However, the fact that this single mutation did not invert the potentiation into inhibition or vice versa on both GlyRs suggests the existence of other molecular sites that determine the final NA-Gly effects.

**Table 1:** Electrophysiological properties of wild-type and mutated GlyRs.

Construct	EC_50_ (µM)	n_H_	I_max_ (pA)	*n*
α_1_ WT	68±1	2.5±0.09	4113±491	8
α_2_ WT	120±6	1.8±0.15	3133±378	7
α_3_ WT	166±8	1.9±0.16	1446±331	12
α_1_α_2_	86±3	2.3±0.14	3781±801	5
α_2_α_1_	111±1	2.4±0.12	4442±845	5
α_1_ I240V/G254A/S296A	37±1*	2.5±0.11	6726±989	5
α_1_ I240V/G254A	32±2*	1.9±0.20	3447±666	5
α_1_ G254A/S296A	73±3	1.9±0.12	3343±990	5
α_1_ I240V	63±1	2.4±0.11	5285±787	5
α_1_ G254A	43±1*	2.5±0.08	5026±578	6
α_1_ S296A	84±1	2.2±0.06	4852±507	5
α_2_ A261G	217±6*	2.1±0.10	3185±678	6
α_3_ A265G	306±4*	2.3±0.05	1873±284	7
α_1_ A52T	195±4*	2.2±0.10	5243±910	5
α_2_ T59A	63±1*	2.2±0.05	3270±678	5
α_2_ T59A/A261G	76±2*	2.2±0.10	3628±1429	5
α_2_ T59A/A261G/A303S	102±2	2.1±0.08	3477±995	5
α_2_ T59A/A261G/A303S/K385A	48±1*	2.2±0.09	4777±1331	6
α_1_ A52T/G254A/S296A	103±4*	2.1±0.15	4957±1214	6
α_1_ K385A	43±3*	2.2±0.24	3474±505	8
α_2_ K385A	76±2*	1.6±0.07	3409±569	8
α_3_ K385A	107±3*	2.5±0.12	2064±575	7

Values are indicated as mean ± s.e.m. from the indicated number of cells.

*indicates significant difference (P<0.05, ANOVA) against the corresponding wild type GlyR subtype.

**Figure 4:**
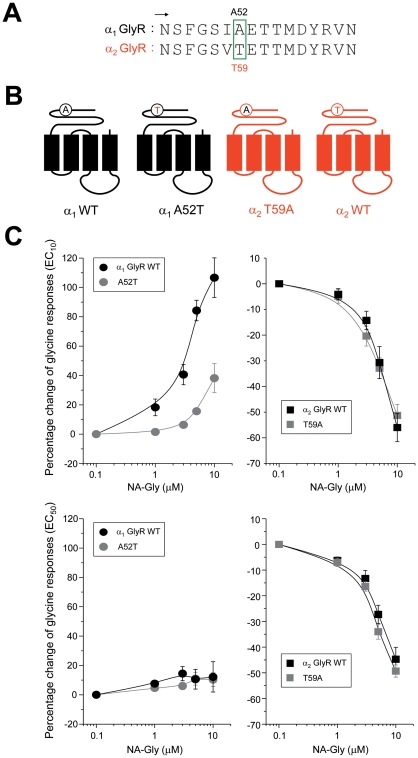
The composition of extracellular loop 2 influences the NA-Gly-induced potentiation of α_1_ GlyRs. (**A**) Amino acid sequence alignment between α_1_ and α_2_ GlyRs within the extracellular loop 2. The A52 residue in α_1_ GlyRs and their homologous position in α_2_ GlyRs are highlighted by a green box. (**B**) Schematic depictions of GlyRs with point mutations in the extracellular loop 2 (**C**) Concentration-response curves for NA-Gly in wild-type and extracellular loop 2-mutated α_1_ and α_2_ GlyRs using two different agonist concentrations. The mutation A52T significantly attenuated the NA-Gly sensitivity of α_1_ GlyRs, whereas the reverse mutation in α_2_ GlyRs (T59A) did not alter NAGly-induced inhibition.

Many of the key residues involved in the coupling of agonist binding to channel gating are located in the N-terminal ECD. Several electrophysiological and molecular modeling studies have postulated that residues within the loops 2 and 7 of the ECD (terminology established by Brejc and coworkers) [30] are critical for the events that precede channel gating [27]. In addition, recent studies have proposed that the generation of a pre-open conformation of the ion channel is a key determinant to explain the differences between full and partial agonists on GlyRs [31]. Interestingly, a specific residue within the extracellular loop 2 of the α_1_ GlyR (A52) has been implicated in the generation of this pre-open conformation [32]–[33], and furthermore, mutation of this amino acid into its α_2_ GlyR counterpart (A52S in human or A52T in rat GlyRs) decreases the sensitivity to the allosteric effects of ethanol [15], [17]. Thus, it is possible that this residue may also contribute to the NA-Gly effects on GlyRs and we directly investigated its importance studying the NA-Gly sensitivity of A52T α_1_ GlyRs and the reverse T59A α_2_ GlyR (Figure 4A). In α_1_ GlyRs, we found that this mutation significantly attenuated positive modulation by NA-Gly, whereas the reverse substitution on α_2_ GlyRs did not change NA-Gly-induced inhibition (Figure 4B). At a higher glycine concentration, these mutations did not significantly affect the receptor sensitivity to this putative EC (Figure 4B). Therefore, these results indicate that only the positive allosteric modulation elicited by NA-Gly on α_1_ GlyRs required the A52 residue. However, these mutations again did not convert the current potentiation into inhibition or vice versa, suggesting that the extracellular and TM domain elements identified here could jointly determine the NA-Gly sensitivity of both GlyR isoforms.

**Figure 5:**
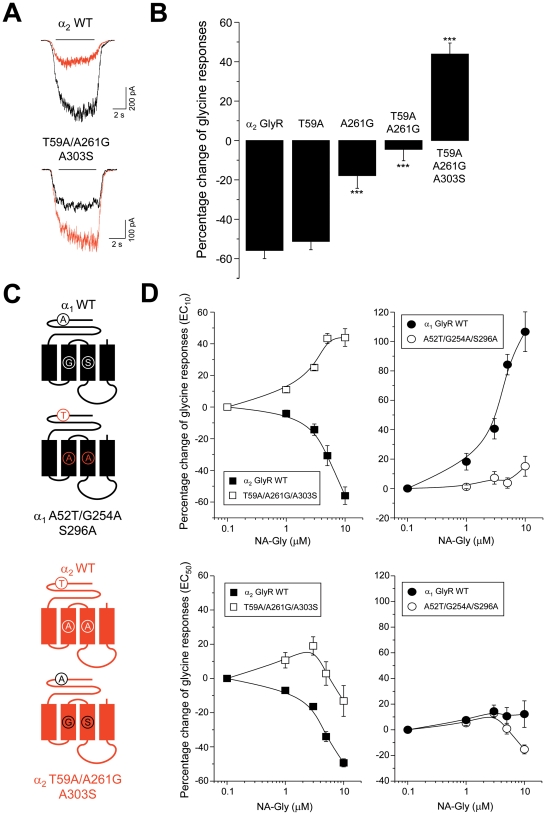
Selective extracellular loop 2 and TM domain mutations in α_2_ GlyRs convert NA-Gly into an allosteric potentiator. (**A**) Examples of glycine-activated current traces from wild-type α_1_ and mutant α_2_ T59A/A261G/A303S GlyRs in the presence of NA-Gly (in red) (**B**) Summary of the effects of NA-Gly after simultaneous extracellular loop 2 and TM reverse mutations on α_2_ GlyRs (*** P<0.001, vs α_2_ GlyRs) (**C**) Schematic diagrams of the triple mutated α_1_ and α_2_ GlyRs (**D**) Sensitivity of the normalized glycine-activated currents elicited in wild-type and triple mutated GlyRs to different concentrations of NA-Gly. Note that three simultaneous reverse mutations in α_2_ GlyR converted NA-Gly into an allosteric potentiator, whereas the homologous substitutions within α_1_ GlyR still did not produce a significant inhibition.

To explore this idea, we studied receptors in which both TM and loop 2 residues were exchanged (Figure 5A–C). These experiments showed that in α_2_ GlyRs, the combined reversal T59A/A261G significantly reduced NA-Gly-induced inhibition to −4±6% (10 µM, n = 10), but still did not change the direction of modulation. Surprisingly, the incorporation of the reversal A303S substitution into this double-mutated GlyR was able to convert NA-Gly from an inhibitor to an allosteric potentiator (44±5%, 10 µM, n = 10) (Figure 5A–C). At a higher glycine concentration, the NA-Gly-induced inhibition on α_2_ GlyRs was also significantly attenuated (Figure 5D). On the other hand, 10 µM of NA-Gly did not inhibit α_1_ GlyRs containing the reverse mutations (A52T/G254A/S296A) at EC_10_ of glycine, but showed a partial inhibition at EC_50_ (Figure 5C). These results suggest that the potentiating effects of NA-Gly on α_1_ GlyRs are mainly determined by the residues A52T/G254A/S296A, whereas the negative effect depends on unidentified molecular determinants only present in α_2_, but not in α_1_ GlyRs.

**Figure 6:**
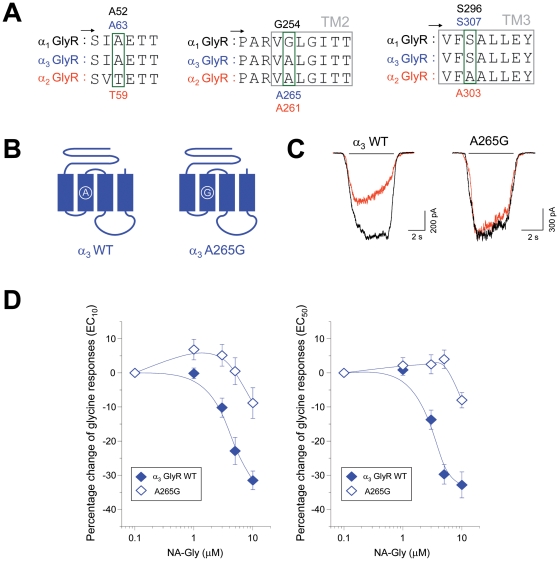
The negative allosteric effects of NA-Gly on α_3_ GlyRs were attenuated but not reverted by altering the TM2 domain composition. (**A**) Primary sequence alignment of α_1_, α_2_ and α_3_ GlyR subunits in selected extracellular loop 2, TM2 and TM3 segments (**B**) Schematic depiction of wild type and point mutated α_3_ GlyRs. (**C**) Examples of current traces through wild-type and point-mutated α_3_ GlyRs in absence (black) or presence of NA-Gly (10 µM, red) (**D**) Concentration-response curves for NA-Gly in wild-type and TM2-mutated α_3_ GlyRs using two different glycine concentrations. Note that this specific mutation A265G in α_3_ GlyRs significantly attenuated the NA-Gly-induced inhibition, but did not convert the inhibition into potentiation

In order to investigate if these molecular determinants can also reverse the inhibitory actions of NA-Gly on α_3_ GlyRs, we first performed amino acid sequence alignments of loop 2 and the critical TM regions. These analyses revealed only one residue, on TM2, which was not conserved between α_1_ and α_3_ GlyRs (G254 in α_1_, A261 in α_2_ and A265 in α_3_, Figure 6A). Taking into account the findings described in Figure 5, it seems fair to suggest that the introduction of the point-mutation A265G in α_3_ GlyRs should be sufficient to convert the NA-Gly-mediated inhibition into a potentiation. Introduction of the A265G mutation in α_3_ GlyR significantly reduced the GlyR current inhibition by NA-Gly (-8±4%, 10 µM, n = 6) but did not convert the inhibition into potentiation (Figure 6B-C). At higher agonist concentrations, this mutation also significantly attenuated NA-Gly-induced inhibition (Figure 6C), indicating a common role of this residue in the negative modulation of α_2_ and α_3_ GlyRs at low and high glycine concentrations (see also Figure 3).

These observations demonstrate that residues in extracellular and TM domains jointly determine the positive modulation of α_1_ GlyRs by NA-Gly, and suggest that acidic ECs could share similar mechanisms of action on these receptors. On the other hand, the current inhibition induced by NA-Gly appears to be largely determined by the nature of the 2′ residue in the TM2 of α_2_ and α_3_ GlyRs (A261 and A265, respectively). However, all these residues appear dispensable for potentiation by hydroxylated ECs (compare Figure 1). Thus, these results indicate that other residues, possibly conserved between the GlyR isoforms, should be critical for the positive allosteric modulation by neutral ECs.

### A basic intracellular residue is critical for the positive endocannabinoid allosteric modulation of GlyRs

**Figure 7:**
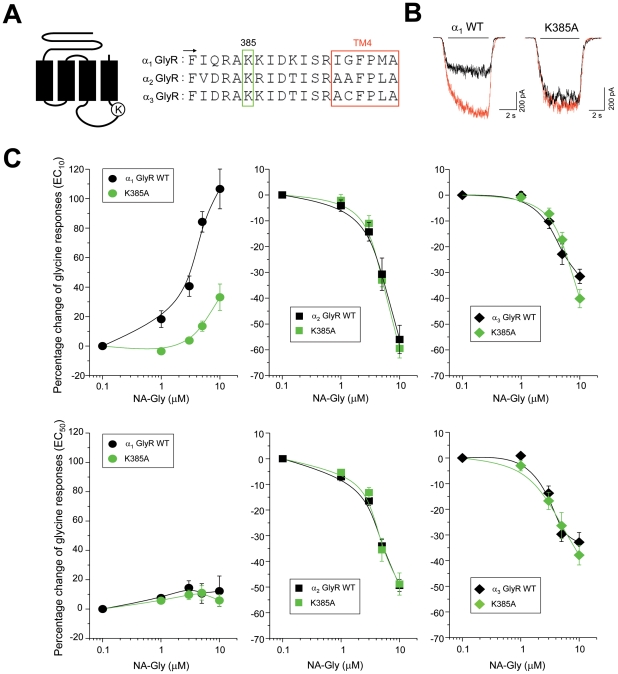
The positive allosteric modulation elicited by NA-Gly is influenced by a conserved lysine residue within the α_1_ GlyR large intracellular loop. (**A**) The schematic receptor representation and the primary sequence alignment describe the position of the conserved intracellular K385 residue within the GlyR structure (**B**) Glycine-activated current traces from wild-type or K385A-mutated α_1_ GlyRs before (black) and during the application of NA-Gly (5 µM, red) (**C**) Concentration-response curves for NA-Gly obtained from wild-type and K385-mutated GlyRs. The intracellular mutation significantly attenuated the NA-Gly-induced potentiation of α_1_ GlyRs.

The results presented above suggest that none of the known ECD and TM molecular sites for allosteric modulators are responsible for the positive modulation by hydroxylated ECs on GlyRs. In addition, since AEA effects on GlyRs appear to be different than those TM elements described for ethanol and general anesthetics (S267 in TM2 domain, see ref. [19]), it appears possible that some conserved key residues outside the TM domains are likely to be responsible for the EC effects on GlyRs. Recent studies have explored the role of the large TM3-4 IL for the Cys-loop ion channel function, intracellular regulation and pharmacology. Electrophysiological studies showed that basic residues within the IL of 5-HT_3_ receptors are critical determinants of single channel conductance [34], whereas other intracellular basic residues on GlyRs are critical for direct G protein βγ interaction with the ion channel [35]. In GlyRs, the same basic residues are required for the allosteric effects of ethanol [36], demonstrating that intracellular residues, besides TM amino acids, can contribute to allosteric modulation of GlyRs. Amino acid sequence alignment of the IL revealed that the most critical lysine residue for Gβγ modulation of α_1_ GlyRs is fully conserved between the three GlyR isoforms (K385 in α_1_ GlyRs, Figure 7A). Thus, in order to examine if this amino acid plays a role in the modulation by ECs, we first tested the sensitivity to NA-Gly of K385A mutated GlyRs at EC_10_ glycine (Figure 7B–C). Our results show that the intracellular K385A mutation significantly attenuated the potentiation by NA-Gly on α_1_ GlyRs to 14±4% (5 µM, n = 8), which is similar to those obtained after mutation of extracellular or TM residues (Figure 3–4). In addition, the homologous intracellular mutations (named K385A relative to α_1_ GlyR sequence) did not significantly alter the current inhibition induced by 10 µM of NA-Gly on α_2_ (−59±4%, n = 8) or α_3_ GlyRs (−40±4%, n = 7). The mutation of this residue furthermore did not alter the NA-Gly inhibitory effects at higher glycine concentrations, indicating a selective effect on the positive allosteric effects on α_1_ GlyRs (Figure 7C). This selectivity was further confirmed in experiments performed in the triple mutated T59A/A261G/A303S α_2_ GlyRs, which are potentiated by NA-Gly (Figure 5). In these receptors, the mutation of the conserved basic lysine residue significantly attenuated the NA-Gly potentiation at EC_10_ glycine (−1±5%, 10 µM, n = 5, Figure S3) but did not alter the modulation at high glycine concentration (Figure S3).

**Figure 8:**
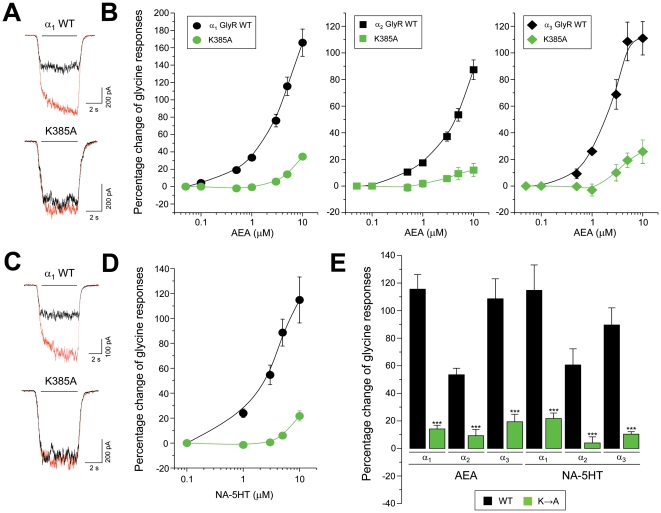
A conserved intracellular lysine residue determines the positive allosteric modulation by neutral ECs on GlyRs. (**A**) Examples of current traces through wild-type α_1_ GlyRs and K385A mutated GlyRs in absence (black) or presence of AEA (5 µM, red) (**B**) Sensitivity to AEA of the normalized glycine-activated currents in wild-type and K385A-mutated GlyRs. The intracellular mutation effectively attenuated the AEA-induced modulation of the three GlyR subunits. (**C**) Glycine-activated current traces from wild-type or K385A-mutated α_1_ GlyRs before (black) and during the application of NA-5HT (5 µM, red) (**D**) Concentration-response curves for NA-5HT obtained from wild-type and K385-mutated α_1_ GlyRs (**E**) Summary of the allosteric effects elicited by AEA and NA-5HT in wild-type and K385A-mutated GlyRs. The current potentiation was significantly attenuated by the intracellular mutation. ***, P<0.001 between each wild-type GlyR and its corresponding K385A mutant.

Because the intracellular lysine residue K385 is conserved between GlyR isoforms and its mutation was able to selectively attenuate the positive allosteric effects of an acidic EC on α_1_ GlyRs and on the triple mutated T59A/A261G/A303S α_2_ GlyRs, it appears likely that this residue could play a critical role in the potentiation elicited by neutral ECs, which is similar between the three subunits examined (Figure 1). We therefore investigated the effects of two hydroxylated ECs on GlyRs with mutations in the K385 residue (Figure 8A-E). The mutated K385A α_1_ GlyR was significantly less potentiated by AEA (14±3%, n = 8, 5 µM). In subsequent experiments we found that the homologous mutations in α_2_ and α_3_ GlyRs also attenuated AEA-induced potentiation (9±5%, n = 7 and 19±5%, n = 6, 5 µM, respectively), demonstrating a critical role for this amino acid in the positive modulation by AEA in all three GlyR subtypes (Figure 8E). Further analyses showed that the positive modulation by NA-5HT, a synthetic EC analog [24], was also significantly attenuated in K385A-mutated α_1,_ α_2_ and α_3_ GlyRs (Figure 8C–E). These data demonstrate that the K385 residue is critical for the positive allosteric modulation of all the GlyR isoforms by both acidic and neutral EC derivatives, but appears to be dispensable for the inhibitory actions of acidic ECs on α_2_ and α_3_ GlyRs.

## Discussion

**Figure 9:**
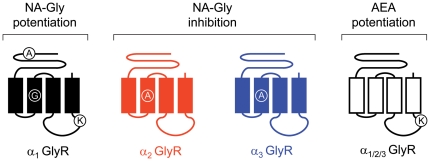
Molecular sites for the allosteric modulation of different GlyR subtypes by ECs. The schematic diagram summarizes the molecular sites for the allosteric modulation of GlyRs by acidic and neutral ECs. The inhibition of α_2_ and α_3_ GlyRs elicited by NA-Gly was specifically influenced by a single TM2 residue (A261 in α_2_ GlyRs and A265 in α_3_ GlyRs), whereas the NA-Gly-induced potentiation of α_1_ GlyRs was reduced by mutating loop 2 (A52), TM2 (G254) or intracellular (K385) amino acids. On the other hand, the AEA-induced potentiation of these three GlyR subtypes was reduced by mutating a conserved intracellular lysine residue (K385 in α_1_ GlyRs).

Previous studies have identified molecular sites for relevant neuromodulators within GlyRs and GABA_A_Rs subunits. Molecular sites for ethanol and volatile anesthetics on α_1_ GlyR have been localized in the interface between the TM2 and TM3 domains, whereas acceptor sites for zinc have been identified in the ECD [12]–[14]. Other reports have identified allosteric sites for etomidate and propofol within the TM2-3 domains of β_2_ and β_3_ GABA_A_Rs subunits and critical residues for neurosteroid effects on TM1 and TM4 regions of α_1_ GABA_A_Rs [27]. Only very recent studies have addressed sites for several cannabinoid ligands on GlyRs. These studies have shown that specific TM residues (S267 and S296 in α_1_ GlyRs) are important for the potentiation elicited by some phytocannabinoids (e.g. Δ^9^-tetrahydrocannabinol (THC) or cannabinodiol) on GlyRs [10], [28]–[29]. Whether these molecular sites affect the cannabinoids actions by interfering with allosteric mechanisms or by affecting their binding is still a matter of debate. Mutations to the S267 residue in α_1_ GlyRs affect the actions of several other allosteric modulators probably by altering their binding [10]–[12], [27]. The importance of this residue for the cannabinoid modulation has been addressed by two groups with conflicting results. While the mutation S267I abolishes the potentiation by cannabidiol and HU210 [28], the S267Q substitution did not change the current enhancement induced by THC or AEA [19]. In this context, the role of the S296 residue in α_1_ GlyRs appears more specific. It has been recently characterized as a pivotal element for THC binding, and furthermore, its mutation did not alter the GlyR sensitivity to other allosteric modulators, such as propofol [29]. In the present study, we have identified residues in the GlyR critical for the positive and negative allosteric modulation by ECs (summarized in Figure 9).

Electrophysiological studies on different wild-type GlyR subtypes identified distinct actions of different ECs, supporting the idea that determinants present on the EC chemical structures plus the existence of specific acceptor sites determine the final allosteric effects [10], [20], [29]. Our results with mutated GlyRs support a role for the A52 and G254 residues in the potentiation of α_1_ GlyRs by acidic ECs. Our finding that successive reverse mutations on the non-conserved extracellular loop 2 and TM residues in α_2_ GlyRs converted the inhibitory effect of NA-Gly into potentiation further supports the idea that the extracellular loop 2 and TM residues are essential elements for the positive allosteric effects of acidic ECs. These results however should be interpreted with some caution. The presence of equivalent loop 2 and TM compositions in α_3_ GlyRs significantly attenuated the NA-Gly inhibitory effect, but did not turn the NA-Gly inhibition into potentiation. In addition, our experiments with mutated α_1_ GlyRs did not show any significant NA-Gly-induced inhibition. Our results thus suggest that the positive and negative actions of acidic ECs on different GlyR subunits are determined by the combination of several molecular sites in each GlyR subunit. Despite the fact that some of these sites are still not identified, it is plausible that the NA-Gly induced inhibition of α_2_ and α_3_ GlyRs occurs through similar mechanisms and molecular sites.

The residues identified in our experiments could affect the acidic EC modulation of GlyRs through different mechanisms. The extracellular loop 2 residue may influence the EC effects by altering the ion channel conformation during pre-open states [31]–[33], whereas the TM residues could either alter putative binding sites [12], [29] or affect the ion channel gating [3]–[5]. Regarding these two TM residues, our data show that only the TM residue at position 2′ within the TM2 helix (G254 on α_1_ GlyR, A261 on α_2_ GlyR, and A265 on α_3_ GlyR) was involved in both NA-Gly-induced potentiation and inhibition. Conversely, the non-conserved TM3 residue (S296 on α_1_ GlyR, A303 on α_2_ GlyR, and A307 on α_3_ GlyR) did not influenced NA-Gly potentiation on α_1_ GlyRs but was necessary to switch the NA-Gly inhibition into potentiation on α_2_ GlyRs. Thus, these findings suggest that the 2′ residue is essential for the allosteric mechanism or for the binding of NA-Gly to the receptor structure, while the TM3 residue likely participates in the allosteric mechanism required for NA-Gly potentiation exclusively on α_2_ GlyRs. Interestingly, a recent report proposed a role for S296 on α_1_ GlyR and A307 on α_3_ GlyRs in the direct binding of THC to TM3 domains possibly via hydrogen bond interactions [29]. Whether the mutations analyzed in our studies preferentially alter acidic EC binding sites or the allosteric mechanisms involved is at present uncertain. However, our results appear to support the fact that part of the positive and negative NA-Gly effects occur in the TM2 region close to the intracellular vicinity of the ion channel pore, whereas loop 2 may regulate these functional modulations through allosteric effects associated to pre-open states of the ion channel.

On the other hand, our results analyzing the potentiation elicited by neutral ECs on GlyRs showed that the non-conserved TM2 and TM3 amino acids between the GlyRs are essentially dispensable. In this context, the pivotal role of the conserved intracellular K385 residue for the positive allosteric effects of both acidic and neutral ECs supports the idea that this residue is essential for the allosteric mechanism behind the GlyR potentiation by ECs. The unchanged NA-Gly-induced inhibition displayed by α_2_ and α_3_ K385A mutants and the lack of inhibition displayed by the reverse α_1_ mutants also suggests that the sites for the positive and negative effects of acidic ECs on GlyRs are different. Based on these results, we propose that the positive EC allosteric site appears to be present in all three GlyR subtypes and is likely to lie in the region between the IL and the TM4 domain in close contact with the lipid-water interface. In contrast, the inhibitory action of acidic ECs appears to be linked related to TM elements present exclusively on α_2_ and α_3_ isoforms and apparently exerts dominance over the positive allosteric site on these GlyR subunits. Together with data from previous studies [3]–[4], [11]–[13], [27], our data suggest that the putative molecular sites for ECs on GlyRs are distinct from previously identified allosteric sites for on GABA_A_ and GlyRs. In addition, our results indicate that the putative molecular TM sites for THC derivatives [28]-[29] and the EC sites on GlyRs are essentially different and apparently unrelated.

There is a large body of evidence to suggest that ECs can elicit CB-R independent actions on ion channels [37]. The Cys-loop family is a particularly well characterized target of ECs [38]. Although our results challenge previous reports in some respects [18], [20], they strongly support the main conclusion of previous reports that ECs may constitute a family of endogenous GlyR modulators with potential impact on the control of neuronal excitability. Our findings on the subunit selectivity and the molecular sites involved provide additional important insights. First, the critical role of the intracellular lysine residue suggests that these effects can be fine tuned by intracellular signaling or post-translational modifications. Intracellular events such as G protein activation [35]–[36], ubiquitination [39]–[40], changes in membrane composition or interactions with accessory proteins may influence the sensitivity of the GlyR subtypes to ECs. Thus, these intracellular events likely contribute to the variability observed in the EC effects on GlyRs. Second, the presence of a modulatory site on the IL matches the intracellular accumulation of ECs reported by others [41]. Several ECs are produced in areas where GlyRs are expressed and their levels also appear to be altered during pathological states, such as spinal cord injury [22], [42]–[44]. Thus, the EC-GlyR interaction could represent a relevant mechanism for the control of inhibitory glycinergic transmission in the spinal cord during pathological conditions, such as chronic pain. Third, the basic character of the key lysine residue and the structure-activity relationship of ECs on GlyRs suggest that the head groups of the hydroxylated and acidic ECs could directly interact with the K385 amino acid. Interestingly, recent studies have highlighted the interaction of TM4 domain with anionic and neutral lipids in other pentameric ligand-gated ion channels [45]–[46]. Moreover, ECs likely acquire an extended conformation in the lipid bilayer with their polar group in close proximity to the membrane phospholipid head groups and the water-lipid interface [47]. Although the data presented here does not provide direct proof, together with the aforementioned reports, we can hypothesize that the lipid surrounded surface of TM4 domains could act as an acceptor of the EC alkyl chains, whereas the IL region that contains the K385 residue could configure the interaction zone with the EC head groups *via* non-covalent interactions. Predicting whether these residues are also directly involved in EC binding is extremely difficult in the absence of crystal structures for GlyRs. It will therefore be interesting to further map the residues within TM4 and IL that may influence the EC allosteric modulation of GlyRs.

In summary, our results provide previously unrecognized molecular sites for the allosteric interaction of ECs with GlyRs. The specific contribution of this interaction to physiology and pathology may in the future become accessible through the generation of gene-targeted mice carrying mutations in the molecular sites identified in the present study. A very recent study has suggested that the positive allosteric modulation exerted by THC on GlyRs is highly relevant for cannabinoid-induced analgesia in animal models of pain, suggesting that several others cannabinoid ligands could represent a promising strategy to develop new pain therapeutics [29]. Thus, a more precise knowledge of the EC sites on GlyRs may in addition provide additional rational basis for the development of novel analgesic drugs acting at specific GlyR subtypes.

## Materials and Methods

### Cell Culture and Transfection

HEK 293 cells (CRL-1573; American Type Culture Collection, Manassas, VA, USA) were cultured using standard methods and were transfected using Lipofectamine LTX (Invitrogen, Carlsbad, CA, USA) with 2 µg of DNA for each GlyR and 0.5 µg of EGFP. Expression of EGFP was used as a marker of successfully transfected cells. All recordings were made 18–36 hours after transfection.

### Electrophysiology

Glycine-evoked currents were recorded from transfected HEK 293 cells in the whole-cell voltage-clamp configuration at room temperature (20–24°C) at a holding potential of −60 mV. Patch electrodes were pulled from borosilicate glass and were filled with (in mM): 120 CsCl, 10 EGTA, 10 HEPES (pH 7.4), 4 MgCl2, 0.5 GTP and 2 ATP. The external solution contained (in mM) 150 NaCl, 10 KCl, 2.0 CaCl2, 1.0 MgCl2, 10 HEPES (pH 7.4), and 10 glucose. Recordings were performed with a HEKA EPC-7 amplifier and Patch Master v2.11 software (HEKA Elektronik, Lambrecht-Pfalz, Germany). The amplitude of the glycine current was obtained using a manually applied pulse (3-6 s) of a sub-saturating glycine concentration (EC_10_ or EC_50_) for each GlyR subunit, using an outlet tube (200 µm ID) of a custom-designed gravity-fed microperfusion system positioned 50–120 µm of the recorded cell. EC_10_ or EC_50_ values for each GlyR studied were obtained experimentally after successive application of 1, 10, 30, 60, 100, 200, 500 and 1000 µM glycine (Table 1). The concentration-response curves parameters (EC_50_ and Hill coefficients, n_h_) were obtained from the curve fits of normalized concentration–response to the equation I_gly_  =  I_max_ (gly)n_h_/((gly)n_h_ + (EC_50_)n_h_). The mean maximal current (I_max_) indicated the average maximal current elicited by a concentration of 1 mM glycine. All modulators were first dissolved in ethanol, DMSO, methanol or methyl-acetate and subsequently diluted into the recording solution on the day of the experiment. None of the vehicles produced discernable effects on the glycine-evoked currents. The drugs were co-applied with glycine, without pre-applications. EC effects were tested up to 10 µM in order to avoid significant membrane-associated effects [48]–[49].

### cDNA Constructs

Mutations were inserted using the QuickChange™ site-directed mutagenesis kit (Stratagene, La Jolla, CA, USA) in cDNA constructs encoding GlyRs in a pCI vector (Promega, Madison, WI, USA) or pcDNA3 (Invitrogen, Carlsbad, CA, USA). All the constructions were confirmed by full sequencing. The procedures involved in the generation of the chimeric GlyRs have been previously published [17]. The GlyR amino acids were numbered according to their position in the mature protein sequence.

### Chemicals

All the drugs were obtained from Tocris Bioscience (Bristol, UK) or BioTrend AG (Zurich, CH). N-arachidonoyl-L-alanine (NALA), N-arachidonoyl-L-serine (NA-Ser) and arachidonic acid (AA) were purchased from Cayman Chemical (Ann Arbor, MI, USA).

### Data analysis

All values were expressed as mean ± s.e.m of normalized glycine-activated currents. Statistical comparisons were performed using ANOVA. P<0.05 was considered statistically significant. For statistical analysis, at least 6 cells were analyzed. For all the statistical analysis and plots, MicroCal Origin 6.0 (Northampton, MA, USA) software was used.

## Supporting Information

Figure S1 **Endocannabinoid sensitivity of different GlyR subtypes.** Concentration-response curves for ECs in wild-type α_1_, α_2_ and α_3_ GlyRs. Membrane currents were activated by equipotent (EC_10_) glycine concentrations. Chemical structures for the ligands are also shown. NOLE, noladin ether; AEA, anandamide; NA-5HT, arachidonyl serotonin; NADA, N-arachidonyl dopamine, NA-Gly; N-arachidonyl glycine; NA-GABA, N-arachidonyl-GABA; NA-Ser, N-arachidonoyl-L-serine; NALA, N-arachidonoyl-L-alanine; AA, arachidonic acid; VIR, virodhamine. Data are means ± SEM from 6-15 cells.
(TIF)

Figure S2 **Concentration-response curves for NA-Gly on wild-type GlyR subunits at different glycine concentrations.** Glycine-evoked currents were activated by glycine at EC_10_ or EC_50_ concentrations and then tested in the presence of NA-Gly. These values were calculated from experimental concentration–response curves (Table 1). Note that the effects of NA-Gly on α_1_ GlyRs were significantly attenuated at a higher glycine concentration, whereas the inhibitory actions of NA-Gly on α_2_ and α_3_ GlyRs were unaffected.
(TIF)

Figure S3 **The potentiation elicited by NA-Gly on the triple mutated α_2_ T59A/A261G/A303S GlyRs also require a lysine residue within the large intracellular loop. (A)** Schematic depiction of wild type and mutated α_2_ GlyRs. **(B)** Concentration-response curves of the normalized glycine-activated currents elicited in wild-type and triple or quadruple mutated α_2_ GlyRs to different concentrations of NA-Gly using an EC_10_ or an EC_50_ of glycine. Three simultaneous reverse mutations in α_2_ GlyR converted NA-Gly into an allosteric potentiator. This current enhancement was significantly attenuated by the mutation of a conserved lysine residue in the large intracellular loop (K385A).
(TIF)
